# COVID-19 Pandemic in Rio de Janeiro, Brazil: A Social Inequality Report

**DOI:** 10.3390/medicina57060596

**Published:** 2021-06-10

**Authors:** Yago Bernardo, Denes do Rosario, Carlos Conte-Junior

**Affiliations:** 1 COVID Research Group, Center for Food Analysis (NAL), Technological Development Support Laboratory (LADETEC), Cidade Universitária, Rio de Janeiro 21941-598, RJ, Brazil; yagoaab@id.uff.br (Y.B.); deneskarosario@ufrj.br (D.d.R.); 2 Graduate Program in Veterinary Hygiene (PPGHV), Faculty of Veterinary Medicine, Fluminense Federal University (UFF), Vital Brazil Filho, Niterói 24230-340, RJ, Brazil; 3 Graduate Program in Food Science (PPGCAL), Institute of Chemistry (IQ), Federal University of Rio de Janeiro (UFRJ), Cidade Universitária, Rio de Janeiro 21941-909, RJ, Brazil; 4 Graduate Program in Sanitary Surveillance, National Institute of Health Quality Control (INCQS), Oswaldo Cruz Foundation (FIOCRUZ), Rio de Janeiro 21040-900, RJ, Brazil

**Keywords:** case-fatality rate, public health, SARS-CoV-2, favelas, socio-economic factors

## Abstract

*Background and Objectives*: To perform a retrospective report on the lethality of COVID-19 in different realities in the city of Rio de Janeiro (RJ). *Materials and Methods*: We accomplished an observational study by collecting the data about total confirmed cases and deaths due to COVID-19 in the top 10 high social developed neighborhoods and top 10 most populous favelas in RJ to determine the case-fatality rate (CFR) and compare these two different realities. *Results*: CFR was significatively higher in poverty areas of RJ, reaching a mean of 9.08% in the most populous favelas and a mean of 4.87% in the socially developed neighborhoods. *Conclusions*: The social mitigation measures adopted in RJ have benefited only smaller portions of the population, excluding needy communities.

## 1. Introduction

The novel coronavirus disease (COVID-19) is a viral illness caused by Severe Acute Respiratory Syndrome Coronavirus 2 (SARS-CoV-2), first diagnosed in Wuhan, China, in December 2019 [1]. Its symptoms include systemic disorders, such as fever, cough, and respiratory disorders, such as pneumonia, similar to previous human beta-coronaviruses [1].

On 5 March 2020, the first report of local transmission was stated in Brazil, and on 20 March 2020, the first death in the city of Rio de Janeiro (RJ) was confirmed. Currently, Brazil occupies a prominent position in the number of deaths by COVID-19, over 200,000 [2]. Among Brazilian states, Rio de Janeiro occupies a central position in this statistic, presenting the second-highest mortality rate [2].

Also, the spread of COVID-19 can be affected by the population’s socio-economic potential, and the city of RJ has significant inequalities in this regard. As the second biggest city in Brazil, with a population exceeding 6 million, about 22% of RJ’s inhabitants live in *favelas* (slums). As described by the Brazilian Ministry of Cities, a favela is a residential area inhabited by low-income families, characterized by precarious living conditions, high population density, and reduced access to essential public services and health care [3]. In addition, another determining factor in the spread of COVID-19 is access to food in the favelas, where about 10% of the population in the favelas could experience hunger during the pandemic [4]. These factors seem to be responsible for higher case-fatality rates (CFR) of endemic viral diseases, such as dengue, for these populations [5]. Kikuti et al. [6] reported higher CFRs were obtained for dengue in locations with higher population density in Brazil.

In this context, this study aimed to perform a retrospective report on the lethality of COVID-19 in different realities in RJ, considering the highest social developed neighborhoods in contrast to the most populated favelas of the city, to understand better the influence of socio-economic factors on the spread of the disease.

## 2. Materials and Methods

### 2.1. Data Collection

The total number of laboratory-confirmed cases and daily deaths by neighborhood and favela were collected from the Rio de Janeiro City Health Secretary [7,8]. The accumulated data was assessed by 22 January 2021. The criterion used to select each neighborhood was the social development index (SDI), described by the Pereira Passos Institute (IPP) [9]. This index was calculated based on several factors, such as (i) adequate water supply, sewage and garbage collection network, (ii) number of bathrooms per resident per household, (iii) illiteracy rate, and (iv) per capita income per household. Favelas were defined as a set of households with at least 51 housing units, irregularly occupying public or private properties, and without access to essential public services [10]. Thus, the top 10 SDI neighborhoods and the top 10 most populous favelas were included to compose this study, as described by the Pereira Passos Institute [9,11].

### 2.2. Epidemiological Index and Statistical Analysis

The CFR between the number of deaths and confirmed cases was assessed as

(1) $$\mathrm{CFR}=\frac{D}{C}$$

wherein D represents the number of deaths, and C represents the number of confirmed cases. The CFR was calculated individually for each neighborhood and favela included in this study.

A one-tailed student’s *t*-test was executed for two independent samples comparing the CFR means between the two groups, at a significance level of 0.05. All analyses were performed using Microsoft Excel 2019^©^ software.

## 3. Results

**Figure 1:**
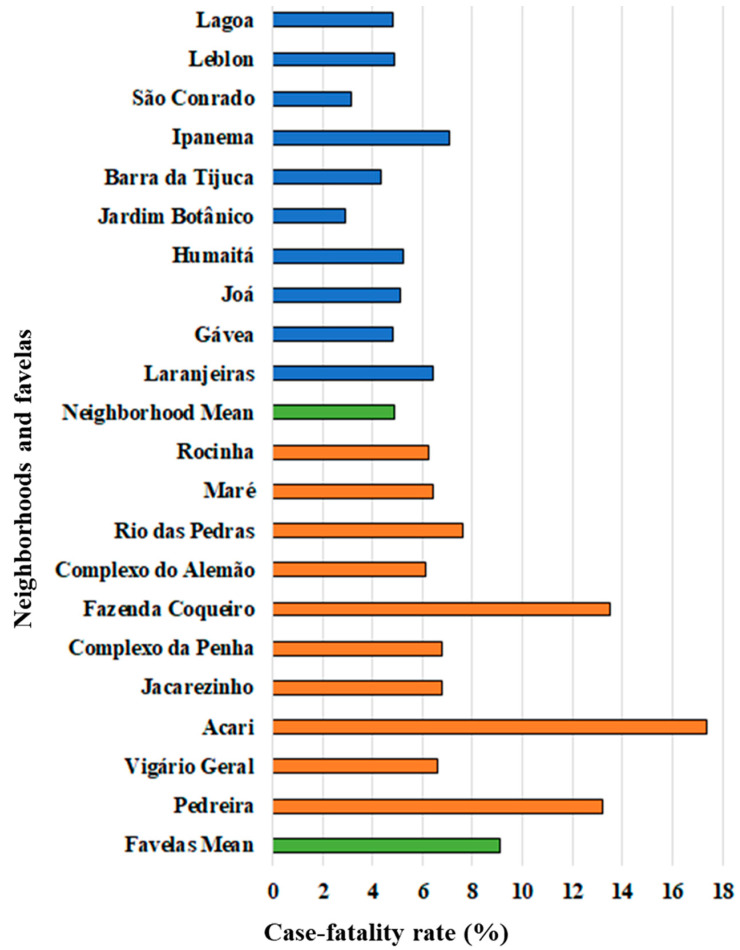
Case-fatality rate (CFR) of COVID-19 in the top 10 highest social developed index (SDI) neighborhoods (blue bars), top 10 most populous favelas of Rio de Janeiro (orange bars), and the average for each scenario (green bars).

The neighborhoods with the highest SDI and the top 10 favelas in RJ, in the number of inhabitants, were presented in Figure 1. The highest CFR was obtained in Ipanema (7.06%) for the neighborhoods, while the lowest CFR was determined in Jardim Botânico (2.94%), and the average presented was 4.87%. In contrast, the average CFR calculated for the favelas of RJ was 9.08%, with the lowest rate being Complexo do Alemão (6.12%) and the highest rate being the favela Acari (17.39%). The difference between CFR averages among neighborhoods and slums was significant (*p* < 0.05).

## 4. Discussion

The results previously described allows us to state that the lethality of COVID-19 in the favelas of RJ is higher when compared to the high social developed neighborhoods. These findings are not surprising and only reiterate the disparities between these two realities in RJ, mainly regarding the availability and access to essential public health services.

One of the main aspects of the hazardous dissemination and lethality of COVID-19 in RJ is poverty. This problem is directly related to food restriction and food quality access, which may be responsible for malnutrition. Food restriction (in quantity and/or quality) can reduce immune response cells, making these individuals more susceptible to transmission and serious clinical injuries from infectious diseases [12]. In addition, the distribution of intensive-care units in RJ presents a high heterogenicity across different areas of the city, with minor coverage in those less privileged, resulting in a greater number of deaths from the severe acute respiratory syndrome in 2020 for low-income families [13]. Also, the disparities in the population distribution reflect the results obtained. The population density of Lagoa (top 1 IDS neighborhood) is 4148 inhabitants per km^2^, while favelas such as Complexo Acari (higher CFR achieved by this study) reach 17,000 inhabitants per km^2^. These numbers imply greater difficulty in implementing social distance measures and lower capacity for medical assistance coverage for this portion of the population.

**Figure 2:**
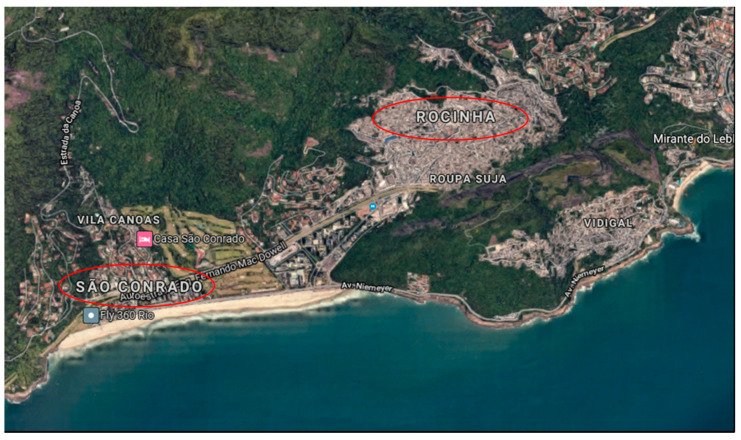
Aerial view of part of the South Zone of Rio de Janeiro. The red ellipses highlight São Conrado and Rocinha. Despite the proximity, Rocinha has twice the CFR of São Conrado (6.27% and 3.12%, respectively). However, its CFR is still lower than the average of the other favelas in Rio de Janeiro (9.08%). Image credits: Google Maps^©^.

On the other hand, although the average CFR presented by the favelas was significantly higher than that shown by the neighborhoods, some favelas gave CFR close to or lower than some neighborhoods. This is the case of Rocinha (6.27%), which had a lower CFR than Laranjeiras (6.42%) and Ipanema (7.06%). A possible explanation for this result would be the proximity of Rocinha to some high SDI neighborhoods, such as São Conrado (Figure 2), both located in the South Zone of RJ. Thus, the availability of medical assistance coverage for the residents of this favela would not be as pronounced as for other favelas, such as Acari (17.39%) and Pedreira (13.20%), both in the North Zone, and Fazenda Coqueiro (13.48%), in the West Zone. On the other hand, the elevated CFR presented by Ipanema, compared to the average of neighborhoods and favelas, can be explained due to the age composition of this neighborhood, where about 28% of the residents are over 60 years old, higher than the average of South Zone (21%), which showed the highest value among the regions of the city [14].

## 5. Conclusions

Rio de Janeiro is one of the main cities affected by the coronavirus but has shown evidence of suppression of mortality from SARS-CoV-2. This behavior is due to follow the WHO recommended control strategies by adopting short-term (e.g., implementation of a lockdown system) and long-term (e.g., early self-isolation, social distancing, remotely medical advice, and efficient support for diagnosis and treatment at home) mitigation measures. However, the data collected and interpreted herein shows that apparently only a small portion of the population has been benefited from these damage mitigation strategies due to unequal access to primary health-care conditions and public policies aimed at the city’s needy communities, aggravated by the COVID-19 pandemic.

## Data Availability

The databases utilized to collect the data presented by this manuscript are available at: https://experience.arcgis.com/experience/38efc69787a346959c931568bd9e2cc4, and https://experience.arcgis.com/experience/8b055bf091b742bca021221e8ca73cd7/, both accessed on 22 January 2021, for the COVID-19 confirmed cases and deaths in Rio de Janeiro neighborhoods and favelas, respectively.
